# Digoxin Dosing Errors, Drug Interactions and Off-Label Risks: Insights from an EudraVigilance Disproportionality Analysis

**DOI:** 10.3390/jcm15155983

**Published:** 2026-07-31

**Authors:** Emilia Sorina Fiat, Laurentiu Stoicescu, Ioana Rada Popa Ilie, Razvan Constantin Vonica, Anca Butuca, Carmen Maximiliana Dobrea, Adina Frum, Claudiu Morgovan, Florina Batar, Crina Cristina Solomon, Felicia Gabriela Gligor

**Affiliations:** 1 Doctoral School, Faculty of Medicine, “Lucian Blaga” University of Sibiu, 550169 Sibiu, Romania; sorina.fiat@yahoo.com; 2 Department of Cardiology, County Clinical Emergency Hospital, 550245 Sibiu, Romania; 3 Department of Cardiology, Vth Medical Clinic, Faculty of Medicine, “Iuliu Haţieganu” University of Medicine and Pharmacy, 400139 Cluj-Napoca, Romania; 4 Department of Endocrinology, Faculty of Medicine, “Iuliu Haţieganu” University of Medicine and Pharmacy, 3-5 Louis Pasteur Street, 400349 Cluj-Napoca, Romania; 5 Preclinical Department, Faculty of Medicine, “Lucian Blaga” University of Sibiu, 550169 Sibiu, Romania; razvanconstantin.vonica@ulbsibiu.ro (R.C.V.); anca.butuca@ulbsibiu.ro (A.B.); carmen.dobrea@ulbsibiu.ro (C.M.D.); adina.frum@ulbsibiu.ro (A.F.); claudiu.morgovan@ulbsibiu.ro (C.M.); florina.batar@ulbsibiu.ro (F.B.); crinacristina.solomon@ulbsibiu.ro (C.C.S.); felicia.gligor@ulbsibiu.ro (F.G.G.)

**Keywords:** inotrope agents, heart failure, pharmacovigilance, EudraVigilance, adverse drug reactions, drug interactions, dosing errors, off-label use

## Abstract

**Background/Objectives**: Digoxin, a positive inotropic agent with a narrow therapeutic window, is used in heart failure but carries toxicity risks. This study aims to characterize the profile of digoxin-associated adverse drug reactions reported in the EudraVigilance database, focusing on drug–drug interactions, dosing errors, and off-label use. **Methods**: Descriptive and disproportionality analyses were performed on Individual Case Safety Reports (ICSRs) until 25 January 2026. Digoxin was compared with other inotropic agents by calculating the Reporting Odds Ratio and 95% confidence intervals. **Results**: Digoxin recorded 11,426 ICSRs, with a predominance in patients aged 65–85 years (46.4%) and over 85 years (25.1%). Higher reporting probabilities were identified for the terms “Drug interaction” and “Overdose” compared to other inotropes. Fatal outcomes were reported in 19 cases related to drug interactions and 41 cases related to overdose. Consistent disproportionality signals were found for gastrointestinal, renal, and nervous system disorders. Off-label use was reported in 0.8% of cases and was associated with unfavorable clinical outcomes. Risks are exacerbated by polypharmacy and renal decline in elderly patients. **Conclusions**: Digoxin toxicity and cardiac complications are the primary drivers of reported morbidity. This risk profile highlights the need for the rigorous clinical monitoring, precise dose adjustments and heightened vigilance regarding drug–drug interactions.

## 1. Introduction

Heart failure (HF) represents a syndrome that is receiving increasing attention from the global medical, academic and clinical communities as epidemiological data show an increased prevalence, while incidence has stabilized over the past decade only in developed countries [1].

Digoxin was one of the first drugs used as a positive inotropic agent [2]. It acts by blocking the sodium–potassium pump in the myocyte membrane, leading to the efflux of intracellular sodium in exchange for calcium, thereby increasing calcium concentration and myocyte contractility. Additionally, it inhibits the atrioventricular node through parasympathetic stimulation, reducing heart rate in patients with advanced HF. It is used in patients with atrial fibrillation (AF) or sinus rhythm (SR). The pivotal DIG study demonstrated that, among patients in sinus rhythm (SR), digoxin reduced hospitalizations but had no significant effect on mortality [3]. Digoxin has a narrow therapeutic window, in which the risk of side effects often outweighs the benefits. Thus, the DIG post hoc analysis suggests potential mortality reduction at low doses (0.5–0.9 ng/mL), an effect that disappears above concentrations of 1 ng/mL [4]. The literature remains contradictory, as several studies show an increased mortality risk in AF patients regardless of HF [5], while others suggest a lower risk of mortality in AF patients but higher for SR ones [6]. However, the use of positive inotropic agents does not always produce the expected effects; this is due to complex pharmacological mechanisms involving drug interactions, altered renal function, tachyphylaxis, QT interval prolongation, cellular calcium overload, energy stress, etc. [7,8,9]. Thus, relatively small variations in digoxin pharmacokinetics can lead to potentially toxic serum concentrations, especially in patients with reduced renal clearance or those experiencing drug interactions. Particular attention should be paid to the elderly, who are at increased risk due to physiological changes and comorbidities [10]. Clinical toxicity may be nonspecific, presenting gastrointestinal and neurological manifestations, conduction disturbances and arrhythmias, which may be severe, thus increasing the risk of toxicity. In this regard, recent studies highlight that acute and chronic toxicity remain relevant events in real-life practice, even though digoxin use has decreased in some health systems [11].

Drug interactions and dosing errors are significant determinants of digoxin-related adverse events. Polypharmacy, common in HF and AF patients, may increase the likelihood of co-administering drugs that may influence digoxin’s absorption, distribution, metabolism or elimination, or potentiate the pharmacodynamic effects on atrioventricular conduction [12].

From a pharmacovigilance perspective and based on real-world data from the European system for the collection and analysis of spontaneous pharmacovigilance reports, EudraVigilance (EV) supports post-authorization surveillance and the safety of digoxin. Spontaneous reports are particularly useful for highlighting risk patterns, especially for situations such as interactions, medication errors or off-label use, which may not be sufficiently detailed or analyzed in randomized clinical trials. Although several pharmacovigilance studies have evaluated digoxin using other spontaneous reporting systems (e.g., FAERS), comparable evidence from the European EV database remains limited. Because EV captures reports from European healthcare systems with different prescribing practices, regulatory frameworks, and reporting patterns, evaluation in this independent database may provide complementary evidence and help assess the consistency and generalizability of previously identified safety signals. Based on these considerations, the present study aims to characterize the profiles of digoxin-associated adverse reactions reported in EV, with emphasis on three practical particularities that have a direct impact on patient safety: drug interactions, dosing errors, and off-label use. In addition, by applying disproportionality analysis and comparing digoxin with other positive inotropic agents included in EudraVigilance., the study aims to identify areas in which digoxin exhibits a distinct reporting profile that is likely to guide prevention, education, and clinical monitoring measures. To the best of our knowledge, this is the first pharmacovigilance study based on real-world EV data to comprehensively evaluate reports of dosing errors, drug interactions, and off-label use associated with digoxin. By characterizing these medication-use issues in a contemporary European spontaneous reporting database, this study provides additional real-world evidence to support pharmacovigilance and safer prescribing practices.

## 2. Materials and Methods

### 2.1. Study Design

The present study was based on publicly available aggregated EV data and therefore focused on disproportionality signals rather than individual case-level analyses. Data included in Individual Case Safety Reports (ICSRs) for reporting spontaneous adverse drug reactions (ADRs) related to digoxin in the EV database (www.adrreports.eu) until 25 January 2026 were used to perform descriptive and disproportionality analyses [13]. Each ICSR contains patients’ personal information (age, sex), origin of reports (the European Economic Area (EEA) or non-EEA) and the reporter category (healthcare professionals—HP, or non-HP) [14]. Aggregated data were extracted between 27 and 29 January 2026.

When reporting ADRs, a hierarchical structure must be used, as defined by the Medical Dictionary for Regulatory Activities (MedDRA). Thus, there are many levels of classification of “medical and health-related” conditions. Therefore, for coding ADRs, MedDRA includes many preferred terms (PTs). These PTs represent unique adverse effects or clinical conditions, and they are subordinated to 27 System Organ Classes (SOCs), which represent the highest level of hierarchy and include PTs grouped according to the affected system [15].

### 2.2. Descriptive Analysis

The descriptive analysis included a comparison of total ICSRs submitted in EV for digoxin with those for other inotropic agents: dobutamine, milrinone, levosimendan, dopamine, epinephrine, and norepinephrine [13]. The comparator group consisted of positive inotropic agents currently used in contemporary clinical practice. Other cardiac glycosides were not selected because of their limited contemporary use, restricted availability, or insufficient numbers of ICSRs in EV to support robust disproportionality analyses. Although these agents differ from digoxin with respect to clinical indication and treatment setting, they represented the most appropriate comparator group with adequate reporting volume.

Regarding the reports for digoxin, general characteristics included in ICSRs were analyzed: age, sex, geographical origin, and reporter [16]. Subsequently, the distribution of digoxin-related cases by severity was analyzed. To compare the severity of ADRs by SOC, a ratio of serious-to-non-serious (RSN) cases was established:

$$RSN=\frac{Number\;of\;serious\;cases}{Number\;of\;\mathrm{non-serious}\;cases}$$

where

RSN = ratio of serious-to-non-serious cases.

To accommodate the wide range of values across SOC categories and improve visualization in a single plot, a logarithmic (Log_10_) bar chart was used.

Another aspect of this analysis was the distribution of cases related to digoxin interactions, dosing errors, and off-label use (Supplementary Materials Table S1), which were subsequently stratified by outcome and, respectively, by reporter category (HP and non-HP). The PTs included in these analyses were selected a priori based on their relevance to the study objectives and according to the MedDRA terminology (Supplementary Materials Table S1). Each PT was analyzed independently as a separate category. Because only aggregated EV data were assessed, it was not possible to determine whether ICSRs contributed to more than one PT category or to evaluate potential overlap between related PTs, such as drug interactions and medication errors. Since unfavorable outcomes included fatal and non-recovered/non-resolved cases (Supplementary Materials Table S2), these data were used to perform a stratified analysis.

### 2.3. Disproportionality Analysis

According to the European Medicine Agency (EMA) regulations, to evaluate disproportionate signals, the Reporting Odds Ratio (ROR) and 95% confidence interval (95% CI) should be calculated. A signal of disproportionate reporting is defined if the lower bound of the 95% confidence interval is greater than 1 and the number of ICSRs is greater than or equal to 5 [17].

Thus, RORs and 95% CIs were calculated for each PT according to EMA recommendations by comparing digoxin with the predefined positive inotropic comparator group [18]. Comparators were selected according to the following predefined criteria: (i) positive inotropic agents; (ii) authorized for use within the European Economic Area (EEA); (iii) currently used in the management of heart failure; and (iv) availability of more than 100 ICSRs in EudraVigilance, to ensure sufficiently robust disproportionality analyses. Accordingly, enoximone (16 ICSRs) was excluded because the limited number of reports was considered insufficient for reliable disproportionality estimates, while digoxin, levosimendan, milrinone, dobutamine, dopamine, epinephrine, and norepinephrine were included. Although these agents differ in their indications, treatment settings, and patient populations, they were selected as the currently available positive inotropic drugs with sufficient pharmacovigilance data to provide contextual interpretations of digoxin reporting patterns rather than direct comparative clinical safety.

Initially, a disproportionality analysis at the SOC level was conducted using the inotropic agents as comparators. Subsequently, disproportionality was evaluated for each eligible PT (*n* > 5) related to digoxin–drug interactions, dosing errors and off-label use [17]. A higher probability of reporting is considered if the case count is ≥5 and the lower limit of the 95% confidence interval exceeds 1.0 [17,19]. The calculation was conducted with MedCalc Software (Version 23.4.8, MedCalc Software Ltd., Ostend, Belgium) on https://www.medcalc.org/calc/odds_ratio.php (accessed on 10 February 2025) [20].

### 2.4. Ethics

This study uses secondary and aggregated data extracted from EV. Because the research involves publicly available, fully anonymized data, and does not involve direct interaction with human subjects, clinical interventions, or access to identifiable personal health information, it is exempt from Ethics Committee approval [21,22].

## 3. Results

### 3.1. Descriptive Analysis

#### 3.1.1. General Characteristics of ICSRs

**Figure 1:**
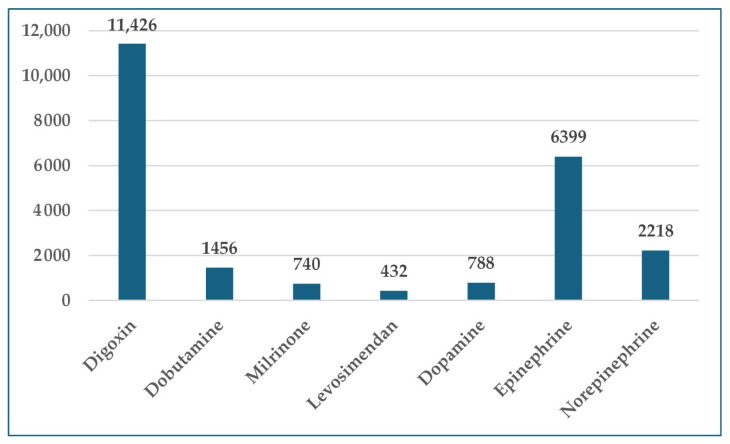
Total ICSRs reported in the EV database up till 25 January 2026.

Our analysis shows that the highest number of ICSRs (*n* = 11,426) in the EV database was reported for digoxin. By comparison, the other inotropic agents had significantly fewer reports (epinephrine: *n* = 6399; norepinephrine: *n* = 2218; dobutamine: *n* = 1456; dopamine: *n* = 788; milrinone: *n* = 740; levosimendan: *n* = 432). The markedly higher number of reports for digoxin is represented in Figure 1.

General characteristics of reports are presented in Table 1. Regarding age distribution, the present analysis highlights the predominance of safety reports in older patients: 65–85 years, 46.4%; more than 85 years, 25.1%. In contrast, ADRs related to dobutamine, milrinone, and levosimendan were more frequently reported in adults aged 18–64 years (29.3–42.4%). This aspect could reflect their typical use in acute settings. Rare cases were reported in the pediatric population (<18 years) across all drugs. Females were more likely to report ADRs with digoxin (57.3%) and epinephrine (55.2%), whereas a balanced sex distribution was observed with milrinone, dobutamine, and norepinephrine. Since levosimendan reports were predominantly originated from the EEA (66.7%), dopamine and dobutamine reports were mostly originated from non-EEA regions. A balanced EEA/non-EEA proportion was noticed for digoxin and norepinephrine. Although the majority of ICSRs were submitted by healthcare professionals for all drugs (digoxin—93.5%, and norepinephrine—97.3%), a notable proportion of reports from non-healthcare professionals were registered for epinephrine (21.6%). This reflects epinephrine use in an emergency or self-administered use.

#### 3.1.2. Distribution of ADR Reports by Seriousness

**Figure 2:**
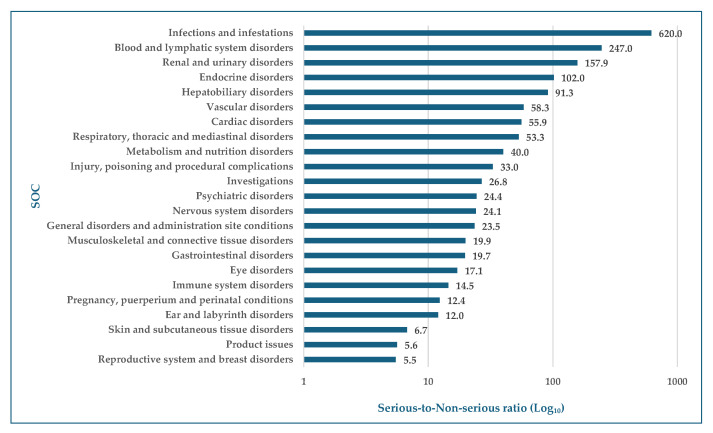
Serious-to-non-serious reports ratio by SOC. The *x*-axis represents the ratio of serious to non-serious reports (RSN) on a logarithmic scale (Log_10_). SOC categories with zero non-serious reports (“Blood and lymphatic system disorders”, “Congenital disorders”, “Social circumstances”, “Surgical and medical procedures”) are not represented in the bar chart.

Our review of EudraVigilance reports for digoxin identified a higher frequency of “Serious” cases than “Non-serious” cases in each SOC (Supplementary Materials Table S3). Thus, the SOC with the highest number of reports is “Cardiac disorders” (*n* = 5143 serious cases, RSN: 55.9), which includes ADR characteristics for digoxin pharmacology (e.g., rhythm disturbances, conduction abnormalities, etc.). In addition to cardiac disorders, other significant serious ADRs were noted in the following SOCs: gastrointestinal disorders (*n* = 2737 serious reports, RSN: 19.7), nervous system disorders (*n* = 2193 serious reports, RSN: 24.1), psychiatric disorders (*n* = 1319 serious reports, RSN: 24.4), and eye disorders (including specific visual disturbances), were documented in 599 serious cases, RSN: 17.1 (Figure 2).

Also, it could be noticed that in some SOCs, a high frequency of ADRs was related to digoxin acute or chronic toxicity: (i) “Injury, poisoning and procedural complications” (*n* = 4458 serious reports, RSN: 33.0), represented by overdosing, medication errors, digoxin poisoning, etc.; (ii) “General disorders and administration site conditions” (*n* = 3673 serious reports, RSN: 23.5) that includes systemic manifestations of drug toxicity; (iii) “Investigations” (*n* = 3002 serious reports, RSN: 26.8) linked to clinical investigations or digoxin monitoring (e.g., high digoxin serum concentration, ECG changes, etc.).

On the other hand, some SOCs with fewer serious reports have a high ratio of serious-to-non-serious cases: “vascular disorders” (*n* = 1107, RSN: 58.3) and “metabolism and nutrition disorders” (*n* = 1802, RSN: 40), suggesting the critical role of electrolyte imbalances in digoxin safety, and “renal and urinary disorders” (*n* = 1263, RSN: 157.9), highlighting the renal clearance route.

#### 3.1.3. Distribution of Interactions, Dosing Errors and Off-Label Use Cases with Unfavorable Outcome

##### Digoxin Interactions

Our study reveals that six PTs reported digoxin–drug interactions, and only two cases had an unfavorable outcome. Thus, 833 reports (7.3%) of the total cases (*n* = 11.426) included drug interactions. Table 2 presents the centralization of cases with an unfavorable outcome. Subsequently, fatal cases were reported in 19 cases related to drug interactions, and 21 cases of drug interactions and 1 case of an inhibitory drug interaction have been declared not recovered or not resolved.

##### Digoxin Dosing Errors

Of the 15 total PTs, most of the digoxin dosing cases were related to overdosing. Only six PT cases included an unfavorable outcome. Thus, from total cases (*n* = 11.426) in 573 cases, the overdose has been reported (4.0%) in 166 cases for accidental overdose (*n* = 166, 1.2%), and 94 cases for intentional overdose (0.7%). Moreover, according to data presented in Table 3, unfavorable outcomes have been reported in 89 cases (41—overdose, 27—accidental overdose, 17—intentional overdose, 2—incorrect dose administered, 1—incorrect dosage administered, 1—incorrect product dosage form administered). On the other hand, not recovered/not resolved cases have been reported in 11 cases of overdose, 8 cases of accidental overdose, 1 case of intentional overdose and 1 case of incorrect dose administered. All these results suggested a higher risk of reporting overdosing compared to underdosing errors. Thus, the type of error influenced the severity of the outcome.

##### Digoxin Off-Label Use

To report digoxin usage outside the Summary of Product Characteristics (SmPC) guidelines, three PTs were used. From total cases (*n* = 11.426), off-label use (*n* = 113) represented 0.8%, product use in an unapproved indication (*n* = 48), 0.3%, and drug effective for an unapproved indication (*n* = 1), 0.0%. Of these, a few cases had unfavorable outcomes: 1 fatal case, 2 cases that were not recovered/not resolved related to off-label use, and 1 case that was not recovered/not resolved related to product use in an unapproved indication (Table 4).

#### 3.1.4. Distribution of ADRs Related to Digoxin Interactions, Dosing Errors or Off-Label Use by Reporter Category

**Figure 3:**
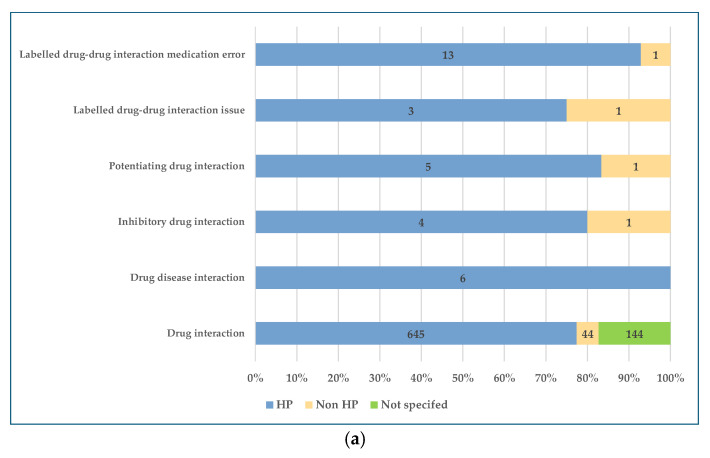

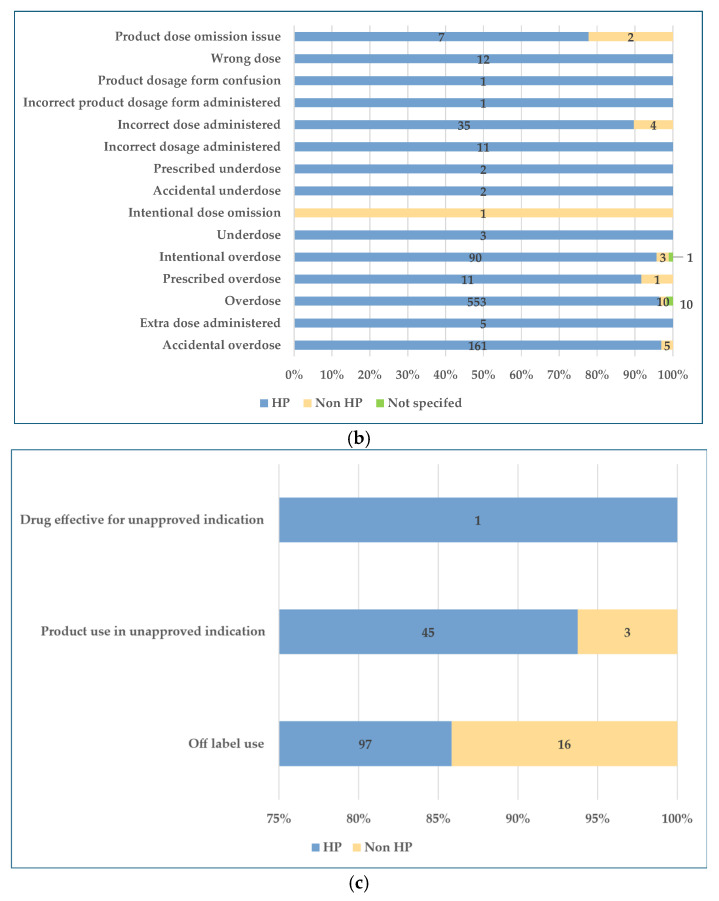
Distribution of digoxin ICSRs by reporter: (**a**)—interactions; (**b**)—dosing errors; (**c**)—digoxin usage outside the SmPC guidelines. HP—healthcare professional.

Figure 3a–c highlights that over 75% of cases related to digoxin interactions, dosing errors or off-label use were reported by healthcare professionals (HP), except intentional dose omission, referring to one case reported by a non-HP (*n* = 1, 0.0%).

### 3.2. Disproportionality Analysis

#### 3.2.1. Disproportionality Analysis by SOC

When digoxin was compared with all inotrope agents included in this analysis, a consistent pattern of disproportionality was identified for several SOCs (Supplementary Materials—Table S4). In Table 5, a higher reporting probability for digoxin was observed than for other inotropes in the following SOCs: gastrointestinal disorders; eye disorders; injury, poisoning, and procedural complications; investigations; metabolic and nutritional disorders; nervous system disorders; psychiatric disorders; and renal and urinary disorders. In contrast, digoxin was markedly associated with a lower reporting probability for vascular disorders compared to all other analyzed medications.

#### 3.2.2. Disproportionality Analysis on PTs Related to Drug–Drug Interactions

**Figure 4:**
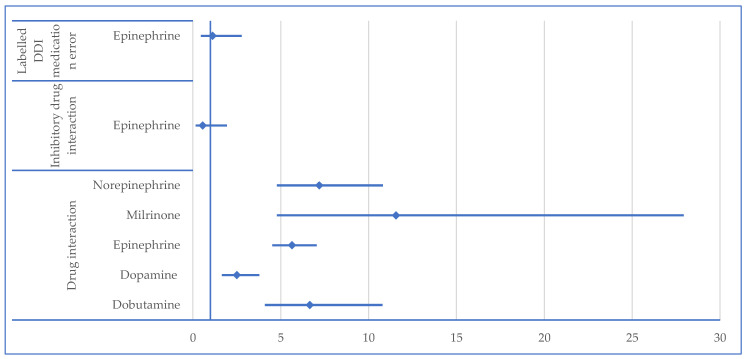
Disproportionality analysis on PTs related to digoxin–drug interactions. DDI—drug–drug interactions.

Figure 4, regarding the event related to drug–drug interactions (DDIs), shows that digoxin was associated with a consistently higher reporting probability for the PT “Drug interaction” when compared to all other comparator drugs used in this analysis. A statistically significant disproportionality was observed for digoxin relative to dobutamine, dopamine, epinephrine, milrinone, and norepinephrine, indicating that digoxin was disproportionately associated with drug interaction reports compared with other agents. On the other hand, no statistically significant disproportionality was identified for the PTs “Inhibitory drug interaction” or “Labeled drug–drug interaction medication error” in the comparison between digoxin and epinephrine, and other predefined DDI-related PTs could not be estimated due to insufficient numbers of reports in the comparator groups.

#### 3.2.3. Disproportionality Analysis on PTs Related to Off-Label Use

**Figure 5:**
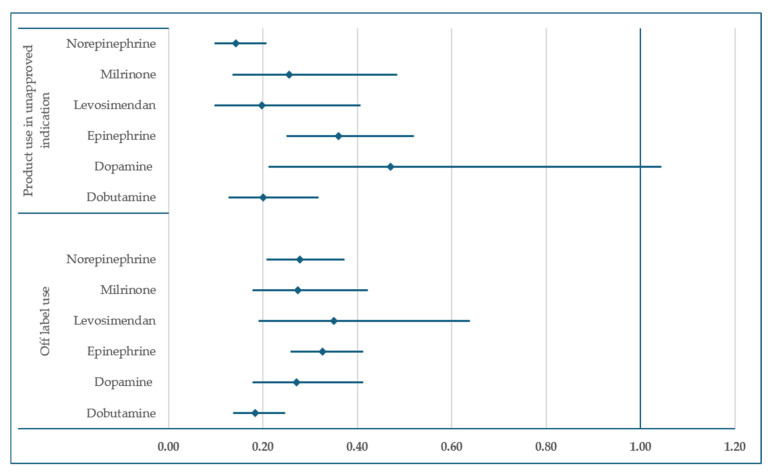
Disproportionality analysis on PTs related to digoxin off-label use.

A similar analysis was performed to evaluate the disproportionate signal of adverse events related to the off-label use of inotropic agents. According to Figure 5, only two PTs had sufficient reports (*n* > 5) to allow meaningful analysis: “Off-label use”, and “Product use in unapproved indication”. For the PT “Off-label use”, digoxin demonstrated a significantly lower reporting probability than all comparator inotropes. RORs ranged for dobutamine (ROR 0.18, 95% CI 0.14–0.25) and levosimendan (ROR 0.35, 95% CI 0.19–0.64). Similar results were observed for “Product use in unapproved indication”: RORs were consistently below 1 for digoxin compared to the other comparators (except dopamine). The lowest disproportionality was observed in comparison to all other comparators (except dopamine). The lowest disproportionality was observed for digoxin compared to norepinephrine (ROR 0.14, 95% CI 0.10–0.21).

#### 3.2.4. Disproportionality Analysis on Dosing Errors PTs

**Figure 6:**
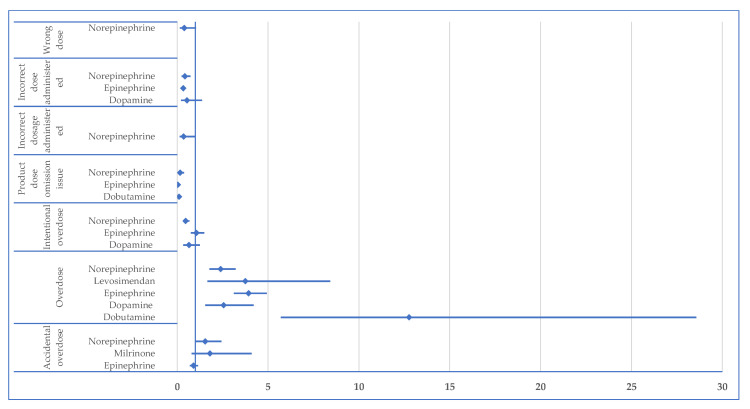
Disproportionality analysis on PTs related to digoxin dosing errors.

In Figure 6, PTs related to digoxin dosing errors were compared to those of other inotropic agents. In terms of general overdose, digoxin demonstrated a disproportionate signal compared to other inotropes. Thus, digoxin had the highest probability of reporting versus dobutamine (ROR 12.76, 95% CI 5.70–28.57), followed by epinephrine (3.92, 95% CI 3.12–4.94), levosimendan (3.75, 95% CI 1.67–8.43), dopamine (2.55, 95% CI 1.54–4.21), and norepinephrine (2.39, 95% CI 1.77–3.22).

For PTs related to dose omission and incorrect administration, digoxin had a lower reporting probability than all drugs used in the study. For example, compared to epinephrine (0.03, 95% CI 0.02–0.06) and dobutamine (0.09, 95% CI 0.04–0.23), digoxin showed a markedly lower probability of reporting product dose omission PTs, whereas, by comparison to norepinephrine, there was a reduced disproportionate signal for incorrect dosage administered (ROR 0.36, 95% CI 0.13–0.96). For intentional overdose, compared with norepinephrine, digoxin showed a reduced likelihood (0.46, 95% CI 0.32–0.68). Also, for accidental overdose or wrong dose related to digoxin, by comparison to all other drugs, no differences could be noticed. Last but not least, for some PTs (e.g., extra dose administered, drug effective for unapproved indication, intentional underdose), the disproportionality could not be calculated because the number of reports among comparator agents was lower than five.

## 4. Discussion

Among inotrope agents, digoxin ranks first in the number of ICSRs reported in EV. A higher number of digoxin reports could reflect its widespread clinical use or extensive pharmacovigilance monitoring [10,23,24]. Additionally, this data provides a relevant basis for performing disproportionality analyses in SOCs and, subsequently, for disproportionality analyses of drug interactions, dosing errors, and off-label use. Our results show a predominance of digoxin reports in older patients (≥65 years). This likely reflects the chronic use of digoxin, especially in this age group, in pathologies such as atrial fibrillation, heart failure, etc. [25,26,27]. Moreover, in these patients, pharmacokinetic changes and increased risk of ADRs are frequently reported [28,29,30]. In contrast, dobutamine, milrinone and levosimendan are used more frequently in emergency conditions, an aspect that could explain the higher frequency of ICSRs reporting these drugs in younger adults [31,32,33,34,35,36].

Regarding the distribution of digoxin ICSRs by sex, a higher proportion was observed in females. These differences could be explained, on the one hand, by drug exposure or different pharmacodynamic behavior [37,38]. Probably because digoxin is a well-known drug [10,39], the origin of ICSRs submitted in EV was similar between both regions, EEA and non-EEA. However, most cases have been reported by healthcare professionals.

Polypharmacy and the renal clearance decline in older patients represent high-risk factors of ADRs related to digoxin use [40,41]. Therefore, to reduce the risk of ADRs and death, a stringent monitoring of these patients is strictly necessary [42,43].

Digoxin interactions have been reported in EV frequently (e.g., 7.3 for drug interactions PTs), in accordance with real-world practice reported in several studies [30,44,45,46]. Moreover, DDIs represent an important component of the risk associated with digoxin use, especially in the context of digoxin’s narrow therapeutic index [11], and taking into consideration the number of fatal cases reported, not least because these may confirm the clinical major relevance of these interactions [47,48,49]. Since labeled interactions (“labeled drug–drug interaction issue” and “labeled drug–drug interaction medication error”) were not related to unfavorable outcomes, it could be suggested that there is better risk recognition, preventive measures already implemented, and a high compliance with the recommendations of the SmPC [50,51].

The present study shows a higher incidence of reports related to overdosing compared to underdosing, with overdose being more clinically relevant based on the pharmacological profile of digoxin. Thus, the narrow therapeutic index contributes to the increasing severity of outcomes [11], and most cases with fatal or non-recovered/non-resolved outcomes have been associated with overdose errors. On the other hand, underdose or omission errors were rarely reported and were not associated with an unfavorable outcome. Although accidental overdose was reported more frequently than intentional overdosing, fatal outcomes occurred at a comparable rate for both PTs (27/166 versus 17/94), suggesting a substantial risk of severe toxicity in both scenarios [52,53,54,55].

These results suggest the need for supplementary measures during prescribing, administration, and patient monitoring, recognizing that dosing errors (including improper dosing) have been associated with unfavorable outcomes (death, not recovered/not resolved). In this regard, digoxin blood levels should be periodically monitored, and dose adjustment should be considered, especially in older patients or those with renal impairment [56,57]. Also, patient and medical staff education is necessary.

Our results also gain additional relevance in light of recent evidence showing that clinically significant digoxin toxicity may occur even when serum concentrations appear to be within the conventional therapeutic range [10,58]. This observation is particularly important for older adults with acute kidney injury, dehydration, hyperkalaemia, or interacting co-medication, in whom tissue sensitivity and rapidly changing pharmacokinetics may make a single laboratory value falsely reassuring [10,12].

On these accounts, the disproportionality signals identified in cardiac, endocrine, metabolic, renal, nervous system, and investigation-related SOCs should be interpreted as interconnected manifestations of the same vulnerability profile rather than isolated events. A clinically meaningful safety strategy therefore requires the combined assessment of symptoms, electrocardiogram abnormalities, serum electrolytes, renal function, and medication review, instead of relying only on digoxin concentration.

The present study suggests that the use of digoxin off-label is rarely reported. However, the occurrence of unfavorable outcomes, including death, indicates that the clinical risk cannot be neglected, especially in the case of its use beyond the approved SmPC indications when the risk of ADRs is increased, particularly given its narrow therapeutic index [11]. These findings highlight the importance of careful benefit–risk assessment when digoxin is used for unapproved indications. Therefore, to minimize risks, off-label use should be exceptional, well justified, and accompanied by close clinical surveillance and monitoring of digoxin plasma levels.

The reports submitted by HP across individual PTs were predominant. This aspect reflects the clinical complexity of digoxin–drug interactions, dosing errors, and use outside the SmPC guidelines. Furthermore, the greater contribution of HP in reporting digoxin ADRs could suggest that physicians are carefully monitoring patients [43,57,59].

On the other hand, the analysis indicates that off-label use of all other inotrope agents is reported disproportionately compared with digoxin, reflecting doctors’ stricter adherence to digoxin’s approved indication in clinical practice. This is probably based on regulatory oversight, a narrower therapeutic index, a high risk of severe ADRs or standardized protocols for using digoxin [60,61,62].

Nevertheless, our analysis indicates that digoxin exhibits a distinct pattern of dosing-related adverse event reports compared with other commonly used inotropes. The higher RORs observed for general overdose generate a pharmacovigilance signal, highlighting the need for further investigation into the potential dosing errors associated with digoxin. In contrast to other inotropes such as epinephrine, norepinephrine, and dopamine, digoxin showed lower disproportionality for the PT product dose omission issue, and, compared to norepinephrine, for the following PTs: “Intentional overdose”, “Incorrect dosage administered” or “Incorrect dose administered”. Overall, these results emphasize the critical need for careful dosing and monitoring of digoxin, particularly given its propensity for serious adverse effects in the context of dosing errors [40,43,50,55].

The present analysis identified SOCs with highly consistent patterns of disproportionality. Some differences in reporting profiles between digoxin and other inotropic agents are highlighted. Thus, digoxin demonstrated a consistently higher probability of reporting events across several SOCs, including gastrointestinal, neurological, psychiatric, metabolic, renal, and ocular disorders. Many of these events are known ADRs associated with digoxin [58,62,63,64].

The disproportionality analysis of DDI-related PTs revealed a robust and consistent signal for the PT “Drug interaction,” with digoxin showing a higher reporting probability than all analyzed inotropic agents. This finding likely reflects digoxin’s well-known susceptibility to clinically relevant drug–drug interactions, attributable to its narrow therapeutic index, dependence on pharmacokinetic or pharmacodynamic modulation and on renal clearance [11,28,29,30]. This may indicate that the increased reporting observed for the broader PT “Drug interaction” predominantly captures nonspecific or clinically suspected interactions.

Other findings of the present study suggest that off-label use reports were less frequently associated with digoxin than with other inotropic medications, probably because of its specific recommendations for worsening or severe heart failure due to left ventricular systolic dysfunction, cardiac failure accompanied by atrial fibrillation and/or supraventricular arrhythmias, particularly chronic atrial flutter and fibrillation [27,49,62,65].

Digoxin showed a higher probability of reporting overdosing errors compared to dobutamine, dopamine, epinephrine, levosimendan, and norepinephrine. These findings could be explained by differences in clinical use. Thus, digoxin is prescribed for chronic conditions with careful therapeutic monitoring, since the other inotropic agents are more frequently in acute conditions or in emergency settings, situations associated with a higher risk of dosing errors [66,67,68,69].

From a pharmacovigilance perspective, these findings also support a more integrated approach to signal interpretation. Linking spontaneous reports with longitudinal healthcare databases, therapeutic drug monitoring data, and prescribing records could better clarify which signals reflect true excess risk, which are amplified by case-mix, and which may be preventable through structured monitoring pathways [70]. Moreover, the present study was designed to characterize pharmacovigilance reporting patterns based on MedDRA-coded reports rather than to identify specific interacting drug pairs or elucidate the underlying mechanisms of drug interactions. Future studies focusing on individual concomitant medications may provide additional mechanistic and clinical insights into digoxin-associated interactions.

### Limitations of the Study

Recent methodological work has emphasized that spontaneous reporting systems are highly valuable for signal generation, but they remain vulnerable to significant bias due to under-reporting, selective reporting, incomplete clinical detail, and temporal distortions related to media attention or regulatory actions [70]. The quality and completeness of spontaneous reports may vary considerably, resulting in missing or inconsistent clinical information that may affect the interpretation of individual safety signals. Moreover, the EV database utilizes a voluntary reporting system open to both healthcare professionals and non-healthcare professionals, such as patients and pharmaceutical manufacturers. Since the relationship between medications and ADRs may be influenced by patient comorbidities and polypharmacy, EV data alone is insufficient to establish direct causality. Because digoxin is primarily prescribed as long-term outpatient therapy, whereas most comparator drugs are administered acutely in hospitalized or critically ill patients, the observed disproportionality may partly reflect indication bias, channeling bias, and differences in reporting practices rather than intrinsic differences in drug safety. Therefore, comparative interpretations should be made with caution.

Although EudraVigilance applies procedures to identify and manage duplicate Individual Case Safety Reports, duplicate reports cannot be completely excluded. Consequently, some adverse drug reactions may have been reported more than once, potentially influencing disproportionality estimates. As with other spontaneous reporting systems, this limitation should be considered when interpreting the findings.

Furthermore, some PTs, particularly those related to drug interactions, are broad and may encompass heterogeneous clinical scenarios, limiting the specificity of the analyses. Moreover, the publicly available EV dataset does not provide sufficient case-level detail to identify the specific concomitant medications involved in reports coded with the MedDRA preferred term “Drug interaction.” Consequently, these signals cannot be interpreted mechanistically or attributed to particular interacting drug combinations, limiting their clinical interpretation. Although each MedDRA preferred term was analyzed independently, the aggregated nature of the publicly available EudraVigilance dataset does not allow for case-level assessments of whether reports contributing to different preferred terms (e.g., drug interaction, overdose, medication error, or off-label use) originated from overlapping or independent Individual Case Safety Reports.

Another limitation arises from confounding related to the environment of drug administration. Hospitalization ensures closer monitoring by healthcare professionals, increasing the likelihood of detecting and reporting an adverse event, especially those of greater impact regarding the omission of molecules for parenteral use than for digoxin, a chronic outpatient medication. Furthermore, some PTs, particularly those related to drug interactions, are broad and may encompass heterogeneous clinical scenarios, limiting the specificity of the analyses. Finally, because EV is a spontaneous reporting database without denominator or exposure data, this study cannot estimate incidence, absolute risk, or the true frequency of prescribing or medication administration errors. Furthermore, disproportionality measures such as the ROR are influenced by reporting patterns and the relative utilization of comparator drugs and therefore should not be interpreted as measures of absolute or comparative risk.

However, because EV does not contain sufficient information on renal function, comorbidities, concomitant medications, disease severity, treatment duration, or exposed populations, these factors could not be adjusted for the present analysis. Consequently, residual confounding remains a major limitation.

For digoxin, this means that the signals detected for drug interactions, dosing errors, and off-label use should be viewed primarily as markers of areas requiring targeted risk-minimization measures, not as direct estimates of incidence. Consequently, prospective research is necessary to properly evaluate these associations.

## 5. Conclusions

The distribution of ADRs indicates that digoxin toxicity and cardiac complications represent the most prominent reporting patterns within the analyzed data. The exclusive signal observed for the generic PT “Drug interaction” likely reflects nonspecific clinical suspicion and coding practices rather than mechanistically defined interactions or documented medication errors, underscoring the importance of cautious interpretations of DDI signals derived from spontaneous reports. However, careful documentation and monitoring of digoxin is needed outside its labeled indications. Overall, the findings highlight digoxin’s distinct reporting profile among inotropes, underlining the importance of ongoing pharmacovigilance and prescriber awareness to ensure patient safety. In this respect, clinicians should maintain vigilance regarding the use according to approved indications, dosing calculations, and potential drug–drug interactions to minimize the risk of digoxin-associated overdose events.

## Figures and Tables

**Table 1:** Descriptive characteristics of ICSRs by medication (*n* and %). EEA—European Economic Area.

Characteristic	Digoxin	Dobutamine	Milrinone	Levosimendan	Dopamine	Epinephrine	Norepinephrine
Total ICSRs	11,426	1456	740	432	788	6399	2218
Age groups							
Not Specified	1366 (11.96%)	282 (19.37%)	198 (26.76%)	102 (23.61%)	132 (16.75%)	1527 (23.86%)	270 (12.17%)
0–1 month	70 (0.61%)	25 (1.72%)	36 (4.86%)	4 (0.93%)	45 (5.71%)	39 (0.61%)	24 (1.08%)
2 months–2 yrs	83 (0.73%)	14 (0.96%)	47 (6.35%)	13 (3.01%)	24 (3.05%)	182 (2.84%)	28 (1.26%)
3–11 yrs	47 (0.41%)	20 (1.37%)	38 (5.14%)	10 (2.31%)	25 (3.17%)	425 (6.64%)	40 (1.80%)
12–17 yrs	37 (0.32%)	32 (2.20%)	22 (2.97%)	10 (2.31%)	27 (3.43%)	316 (4.94%)	70 (3.16%)
18–64 yrs	1651 (14.45%)	617 (42.38%)	217 (29.32%)	150 (34.72%)	295 (37.44%)	2943 (45.99%)	1108 (49.95%)
65–85 yrs	5300 (46.38%)	445 (30.56%)	162 (21.89%)	133 (30.79%)	217 (27.54%)	898 (14.03%)	626 (28.22%)
>85 yrs	2872 (25.14%)	21 (1.44%)	20 (2.71%)	10 (2.32%)	23 (2.91%)	69 (1.09%)	52 (2.36%)
Sex							
Female	6550 (57.33%)	584 (40.11%)	300 (40.54%)	133 (30.79%)	326 (41.37%)	3533 (55.21%)	986 (44.45%)
Male	4146 (36.29%)	655 (44.99%)	380 (51.35%)	239 (55.32%)	398 (50.51%)	2485 (38.83%)	1109 (50.00%)
Not Specified	730 (6.38%)	217 (14.90%)	60 (8.11%)	60 (13.89%)	64 (8.12%)	381 (5.95%)	123 (5.55%)
Region							
EEA	5866 (51.34%)	448 (30.77%)	157 (21.22%)	288 (66.67%)	195 (24.75%)	2236 (34.94%)	1105 (49.82%)
Non-EEA	5560 (48.66%)	1007 (69.16%)	583 (78.78%)	144 (33.33%)	592 (75.13%)	4163 (65.06%)	1113 (50.18%)
Not Specified	0 (0.00%)	1 (0.07%)	0 (0.00%)	0 (0.00%)	1 (0.12%)	0 (0.00%)	0 (0.00%)
Reporter							
Healthcare professional	10,682 (93.49%)	1369 (94.02%)	687 (92.84%)	365 (84.49%)	745 (94.54%)	4970 (77.67%)	2159 (97.34%)
Non-healthcare professional	508 (4.45%)	71 (4.88%)	50 (6.76%)	9 (2.08%)	33 (4.19%)	1380 (21.57%)	46 (2.07%)
Not Specified	236 (2.06%)	16 (1.10%)	3 (0.40%)	58 (13.43%)	10 (1.27%)	49 (0.76%)	13 (0.59%)

**Table 2:** Cases of digoxin interactions with unfavorable outcomes.

PT	Total RA	Proportion of Total Cases (*n* = 11.426)	Unfavorable Outcomes	
			Fatal	Not Recovered/Not Resolved
Drug interaction	833	7.3%	19	21
Inhibitory drug interaction	5	0.0%	0	1
Potentiating drug interaction	6	0.1%	0	0
Labeled drug–drug interaction issue	4	0.0%	0	0
Labeled drug–drug interaction medication error	14	0.1%	0	0

**Table 3:** Cases of digoxin dosing errors with unfavorable outcomes.

PT	Total RA	Proportion of Total Cases (*n* = 11.426)	Unfavorable Outcomes	
			Fatal	Not Recovered/Not Resolved
Accidental overdose	166	1.2%	27	8
Extra dose administered	5	0.0%	0	0
Overdose	573	4.0%	41	11
Prescribed overdose	12	0.1%	0	0
Intentional overdose	94	0.7%	17	1
Intentional dose omission	1	0.0%	0	0
Accidental underdose	2	0.0%	0	0
Prescribed underdose	2	0.0%	0	0
Product dose omission issue	9	0.1%	0	0
Underdose	3	0.0%	0	0
Incorrect dosage administered	11	0.1%	1	0
Incorrect dose administered	39	0.3%	2	1
Incorrect product dosage form administered	1	0.0%	1	0
Product dosage form confusion	1	0.0%	0	0
Wrong dose	12	0.1%	0	0

**Table 4:** Cases of digoxin off-label use with unfavorable outcomes.

PT	Total RA	Proportion of Total Cases (*n* = 11.426)	Unfavorable Outcomes	
			Fatal	Not Recovered/Not Resolved
Drug effective for unapproved indication	1	0.0%		
Off-label use	113	0.8%	1	2
Product use in unapproved indication	48	0.3%		1

**Table 5:** Qualitative disproportionality patterns by SOC (classification based on Reporting Odds Ratio and 95% confidence interval).

System Organ Class (SOC)	Digoxin– Dobutamine	Digoxin– Milrinone	Digoxin– Levosimendan	Digoxin– Dopamine	Digoxin– Epinephrine	Digoxin– Norepinephrine
Blood and lymphatic system disorders	⚪	🟢	⚪	⚪	🔴	⚪
Cardiac disorders	🟢	🔴	⚪	🔴	🔴	🔴
Congenital, familial and genetic disorders	🟢	🟢	⚪	🟢	⚪	🟢
Ear and labyrinth disorders	🔴	⚪	⚪	⚪	⚪	⚪
Endocrine disorders	⚪	⚪	⚪	⚪	🔴	🔴
Eye disorders	🔴	🔴	🔴	🔴	🔴	🔴
Gastrointestinal disorders	🔴	🔴	🔴	🔴	🔴	🔴
General disorders and administration site conditions	🟢	🔴	⚪	🟢	🟢	🟢
Hepatobiliary disorders	⚪	⚪	🟢	⚪	🔴	⚪
Immune system disorders	🟢	🟢	⚪	🟢	🟢	🟢
Infections and infestations	⚪	⚪	⚪	🟢	🔴	🟢
Injury, poisoning and procedural complications	🔴	🔴	🔴	🔴	🔴	🔴
Investigations	🔴	🔴	🔴	🔴	🔴	🔴
Metabolism and nutrition disorders	🔴	🔴	🔴	🔴	🔴	🔴
Musculoskeletal and connective tissue disorders	⚪	🔴	⚪	🔴	⚪	⚪
Neoplasms benign, malignant and unspecified	⚪	⚪	⚪	⚪	🔴	⚪
Nervous system disorders	🔴	🔴	🔴	🔴	🔴	🔴
Pregnancy, puerperium and perinatal conditions	⚪	⚪	⚪	🟢	⚪	🟢
Product issues	⚪	🟢	⚪	⚪	🟢	🟢
Psychiatric disorders	🔴	🔴	🔴	🔴	🔴	🔴
Renal and urinary disorders	🔴	🔴	🔴	🔴	🔴	🔴
Reproductive system and breast disorders	⚪	⚪	⚪	⚪	🔴	🔴
Respiratory, thoracic and mediastinal disorders	⚪	🔴	⚪	⚪	⚪	⚪
Skin and subcutaneous tissue disorders	⚪	⚪	⚪	🟢	🟢	🟢
Social circumstances	⚪	⚪	⚪	⚪	🔴	⚪
Surgical and medical procedures	⚪	⚪	⚪	🔴	🔴	⚪
Vascular disorders	🟢	🟢	🟢	🟢	🟢	🟢

🔴 Higher reporting probability; 🟢 lower reporting probability; ⚪ no statistically significant difference.

## Data Availability

The original contributions presented in this study are included in the article/Supplementary Material. Further inquiries can be directed to the corresponding authors.

## Supplementary Materials

The following supporting information can be downloaded at: https://www.mdpi.com/article/10.3390/jcm15155983/s1, Table S1: The preferred term used for descriptive analysis; Table S2: Classification of clinical outcomes; Table S3: Distribution of reports in SOCs by seriousness; Table S4: Quantitative disproportionality patterns by SOC (classification based on Reporting Odds Ratio and 95% confidence interval); Table S5: Quantitative disproportionality patterns by PT (classification based on Reporting Odds Ratio and 95% confidence interval).

## Abbreviations

The following abbreviations are used in this manuscript:

ADR	Adverse Drug Reaction
AF	Atrial Fibrillation
CI	Confidence Interval
DDI	Drug–drug Interaction
EEA	European Economic Area
EMA	European Medicine Agency
EV	EudraVigilance
HF	Heart Failure
HFpEF	Heart Failure with Preserved Ejection Fraction
HFrEF	Heart Failure with Reduced Ejection Fraction
HP	Healthcare Professional
ICSR	Individual Case Safety Report
MedDRA	Medical Dictionary for Regulatory Activities
PT	Preferred Term
ROR	Reporting Odds Ratio
RSN	Ratio of Serious-To-Non-Serious
SmPC	Summary of Product Characteristics
SOC	System Organ Classes
SR	Sinus Rhythm
T3	Triiodothyronine
