# Supplementary material for: Digoxin Dosing Errors, Drug Interactions and Off-Label Risks: Insights from an EudraVigilance Disproportionality Analysis
Source: J Clin Med. 2026 Jul 31;15(15):5983. doi: 10.3390/jcm15155983 (PMC13467352; doi:10.3390/jcm15155983)
Supplement: Supplementary file 1 [file jcm-15-05983-s001.zip › jcm-4434099-supplementary.pdf]

Supplementary Material

Digoxin Dosing Errors, Drug Interactions and Off-Label Risks: Insights from an EudraVigilance Disproportionality Analysis

Emilia Sorina Fiat, Laurentiu Stoicescu *, Ioana Rada Popa Ilie *, Razvan Constantin Vonica, Anca Butuca, Carmen Maximiliana Dobrea, Adina Frum, Claudiu Morgovan, Florina Batar, Crina Cristina Solomon, Felicia Gabriela Gligor

* Correspondence: laurentiu.stoicescu@umfcluj.ro; ioana.ilie@umfcluj.ro

Table S1. The preferred term used for descriptive analysis

Health condition	PT
Drug-drug interaction	Drug interaction
	Inhibitory drug interaction
	Labelled drug-disease interaction issue
	Labelled drug-disease interaction medication error
	Labelled drug-food interaction issue
	Labelled drug-food interaction medication error
	Potentiating drug interaction
	Labelled drug-drug interaction issue
	Labelled drug-drug interaction medication error
Dossing errors	Accidental overdose
	Extra dose administered
	Overdose
	Prescribed overdose
	Intentional dose omission
	Intentional overdose
	Intentional underdose
	Accidental underdose
	Drug dose omission by device
	Incomplete dose administered
	Prescribed underdose
	Product dose omission
	Product dose omission in error
	Product dose omission issue
	Underdose
	Dose calculation error
	Dose calculation error associated with device
	Incorrect dosage administered
	Incorrect dose administered
	Incorrect dose administered by device
	Incorrect dose administered by product

Health condition	PT
	Incorrect product dosage form administered
	Product dosage form confusion
	Wrong dose
Off-label use	Drug effective for unapproved indication
	Off label use
	Off label use of device
	Product use in unapproved therapeutic environment
	Product use in unapproved indication
	Product used for unknown indication
	Unintentional use for unapproved indication

Table S2. Classification of clinical outcomes

Type of outcome	Outcome
Unfavorable	Fatal
	Not recovered/Not resolved
Favorable	Recovered/Resolved
	Recovered/Resolved with sequelae
	Recovering/Resolving
Unspecified	Not specified
	Unknown

Table S3. Distribution of reports in SOCs by seriousness.

SOC	Serious	Non serious	Not specified	Total
Cardiac disorders	5,143	92	9	5244
Injury, poisoning and procedural complications	4,458	135	2	4595
General disorders and administration site conditions	3,673	156	3	3832
Investigations	3,002	112	1	3115
Gastrointestinal disorders	2,737	139	3	2879
Nervous system disorders	2,193	91	7	2291
Metabolism and nutrition disorders	1,802	45	3	1850
Psychiatric disorders	1,319	54	3	1376
Respiratory, thoracic and mediastinal disorders	1,333	25	0	1358
Renal and urinary disorders	1,263	8	0	1271
Vascular disorders	1,107	19	1	1127
Skin and subcutaneous tissue disorders	606	90	1	697
Eye disorders	599	35	0	634
Infections and infestations	620	1	0	621
Musculoskeletal and connective tissue disorders	518	26	1	545

SOC	Serious	Non serious	Not specified	Total
Blood and lymphatic system disorders	494	2	2	498
Social circumstances	360	0	0	360
Surgical and medical procedures	333	0	0	333
Hepatobiliary disorders	274	3	1	278
Pregnancy, puerperium and perinatal conditions	198	16	0	214
Reproductive system and breast disorders	132	24	1	157
Ear and labyrinth disorders	144	12	0	156
Neoplasms benign, malignant and unspecified (incl cysts and polyps)	131	0	0	131
Endocrine disorders	102	1	1	104
Congenital, familial and genetic disorders	81	0	0	81
Immune system disorders	58	4	0	62
Product issues	45	8	0	53

Table S4. Quantitative disproportionality patterns by SOC (Classification based on Reporting Odds Ratio and 95% confidence interval)

**SOC**	**Number of cases reported for digoxin**	Digoxin - Dobutamine	Digoxin - Milrinone	Digoxin - Levosimedan	Digoxin - Dopamine	Digoxin - Norepunephrine	Digoxin - Epinephrine
Blood and lymphatic system disorders	498	ROR: 0.82, 95% CI: 0.64–1.04	ROR: 0.39, 95% CI: 0.30–0.50	ROR: 1.18, 95% CI: 0.71–1.97	ROR: 1.01, 95% CI: 0.71–1.44	ROR: 0.85, 95% CI: 0.69–1.05	ROR: 4.12, 95% CI: 3.20–5.30
Cardiac disorders	5244	ROR: 0.81, 95% CI: 0.72–0.90	ROR: 1.58, 95% CI: 1.35–1.84	ROR: 1.09, 95% CI: 0.90–1.32	ROR: 1.75, 95% CI: 1.50–2.04	ROR: 2.15, 95% CI: 1.94–2.37	ROR: 2.64, 95% CI: 2.47–2.83
Congenital, familial and genetic disorders	81	ROR: 0.20, 95% CI: 0.14–0.29	ROR: 0.47, 95% CI: 0.25–0.89	NA	ROR: 0.46, 95% CI: 0.25–0.85	ROR: 0.37, 95% CI: 0.25–0.54	ROR: 1.08, 95% CI: 0.74–1.57
Ear and labyrinth disorders	156	ROR: 2.00, 95% CI: 1.05–3.80	NA	NA	ROR: 2.17, 95% CI: 0.89–5.30	NA	ROR: 1.05, 95% CI: 0.81–1.38
Endocrine disorders	104	ROR: 1.48, 95% CI: 0.75–2.92	NA	ROR: 0.65, 95% CI: 0.28–1.49	ROR: 0.71, 95% CI: 0.37–1.37	ROR: 2.03, 95% CI: 1.06–3.89	ROR: 2.79, 95% CI: 1.74–4.46
Eye disorders	634	ROR: 4.97, 95% CI: 3.06–8.08	ROR: 4.29, 95% CI: 2.29–8.05	ROR: 4.17, 95% CI: 1.86–9.37	ROR: 2.51, 95% CI: 1.56–4.04	ROR: 4.28, 95% CI: 2.96–6.20	ROR: 1.55, 95% CI: 1.33–1.81
Gastrointestinal disorders	2879	ROR: 3.30, 95% CI: 2.75–3.95	ROR: 13.51, 95% CI: 8.45–21.61	ROR: 4.86, 95% CI: 3.31–7.15	ROR: 4.01, 95% CI: 3.08–5.23	ROR: 3.72, 95% CI: 3.18–4.36	ROR: 4.38, 95% CI: 3.95–4.86
General disorders and administration site conditions	3832	ROR: 0.86, 95% CI: 0.76–0.96	ROR: 1.59, 95% CI: 1.34–1.89	ROR: 1.00, 95% CI: 0.81–1.22	ROR: 0.55, 95% CI: 0.48–0.64	ROR: 0.67, 95% CI: 0.61–0.73	ROR: 0.65, 95% CI: 0.61–0.69

Hepatobiliary disorders	278	ROR: 1.01, 95% CI: 0.71–1.45	ROR: 0.78, 95% CI: 0.50–1.20	ROR: 0.54, 95% CI: 0.34–0.87	ROR: 0.73, 95% CI: 0.49–1.10	ROR: 0.77, 95% CI: 0.59–1.00	ROR: 3.77, 95% CI: 2.72–5.23
Immune system disorders	62	ROR: 0.11, 95% CI: 0.08–0.15	ROR: 0.44, 95% CI: 0.22–0.90	NA	ROR: 0.12, 95% CI: 0.08–0.19	ROR: 0.15, 95% CI: 0.10–0.20	ROR: 0.06, 95% CI: 0.05–0.08
Infections and infestations	621	ROR: 0.94, 95% CI: 0.74–1.19	ROR: 0.81, 95% CI: 0.60–1.09	ROR: 0.94, 95% CI: 0.62–1.41	ROR: 0.55, 95% CI: 0.43–0.71	ROR: 0.51, 95% CI: 0.44–0.60	ROR: 1.47, 95% CI: 1.26–1.71
Injury, poisoning and procedural complications	4595	ROR: 4.77, 95% CI: 4.06–5.60	ROR: 4.68, 95% CI: 3.75–5.83	ROR: 3.42, 95% CI: 2.64–4.42	ROR: 2.68, 95% CI: 2.24–3.21	ROR: 1.77, 95% CI: 1.60–1.96	ROR: 1.28, 95% CI: 1.20–1.36
Investigations	3115	ROR: 1.57, 95% CI: 1.37–1.80	ROR: 2.37, 95% CI: 1.91–2.94	ROR: 2.42, 95% CI: 1.83–3.20	ROR: 1.86, 95% CI: 1.54–2.26	ROR: 2.09, 95% CI: 1.85–2.37	ROR: 3.12, 95% CI: 2.85–3.41
Metabolism and nutrition disorders	1850	ROR: 2.83, 95% CI: 2.28–3.51	ROR: 2.31, 95% CI: 1.76–3.05	ROR: 5.77, 95% CI: 3.38–9.85	ROR: 3.12, 95% CI: 2.30–4.21	ROR: 1.97, 95% CI: 1.69–2.30	ROR: 6.02, 95% CI: 5.18–6.99
Musculoskeletal and connective tissue disorders	545	ROR: 0.87, 95% CI: 0.69–1.11	ROR: 5.24, 95% CI: 2.48–11.10	ROR: 1.75, 95% CI: 0.98–3.13	ROR: 1.67, 95% CI: 1.09–2.54	ROR: 1.20, 95% CI: 0.95–1.51	ROR: 1.01, 95% CI: 0.88–1.17
Neoplasms benign, malignant and unspecified (incl cysts and polyps)	131	ROR: 0.79, 95% CI: 0.50–1.26	NA	NA	ROR: 1.82, 95% CI: 0.74–4.45	ROR: 1.21, 95% CI: 0.76–1.93	ROR: 4.35, 95% CI: 2.62–7.22
Nervous system disorders	2291	ROR: 1.54, 95% CI: 1.32–1.80	ROR: 5.05, 95% CI: 3.59–7.12	ROR: 3.92, 95% CI: 2.63–5.84	ROR: 1.61, 95% CI: 1.31–1.99	ROR: 2.31, 95% CI: 2.00–2.68	ROR: 1.33, 95% CI: 1.22–1.44
Pregnancy, puerperium and perinatal conditions	214	ROR: 1.24, 95% CI: 0.80–1.94	ROR: 0.99, 95% CI: 0.57–1.71	ROR: 1.63, 95% CI: 0.67–3.98	ROR: 0.52, 95% CI: 0.35–0.77	ROR: 0.55, 95% CI: 0.42–0.71	ROR: 1.02, 95% CI: 0.81–1.27

Product issues	53	ROR: 0.67, 95% CI: 0.34–1.33	ROR: 0.09, 95% CI: 0.06–0.14	ROR: 0.40, 95% CI: 0.16–1.00	ROR: 0.52, 95% CI: 0.24–1.15	ROR: 0.19, 95% CI: 0.13–0.27	ROR: 0.02, 95% CI: 0.01–0.02
Psychiatric disorders	1376	ROR: 2.35, 95% CI: 1.87–2.97	ROR: 5.20, 95% CI: 3.28–8.22	ROR: 11.69, 95% CI: 4.83–28.29	ROR: 2.78, 95% CI: 1.99–3.88	ROR: 3.16, 95% CI: 2.55–3.93	ROR: 2.63, 95% CI: 2.32–2.98
Renal and urinary disorders	1271	ROR: 2.21, 95% CI: 1.75–2.80	ROR: 1.62, 95% CI: 1.22–2.16	ROR: 2.04, 95% CI: 1.35–3.06	ROR: 1.35, 95% CI: 1.04–1.74	ROR: 1.43, 95% CI: 1.22–1.69	ROR: 4.40, 95% CI: 3.75–5.17
Reproductive system and breast disorders	157	ROR: 1.43, 95% CI: 0.83–2.49	NA	NA	ROR: 2.18, 95% CI: 0.89–5.33	ROR: 1.80, 95% CI: 1.09–2.98	ROR: 4.04, 95% CI: 2.58–6.32
Respiratory, thoracic and mediastinal disorders	1358	ROR: 0.90, 95% CI: 0.77–1.06	ROR: 1.56, 95% CI: 1.19–2.04	ROR: 1.01, 95% CI: 0.75–1.36	ROR: 1.00, 95% CI: 0.80–1.24	ROR: 1.06, 95% CI: 0.92–1.22	ROR: 0.98, 95% CI: 0.89–1.07
Skin and subcutaneous tissue disorders	697	ROR: 0.92, 95% CI: 0.74–1.15	ROR: 1.44, 95% CI: 1.00–2.07	ROR: 1.27, 95% CI: 0.81–1.98	ROR: 0.68, 95% CI: 0.52–0.88	ROR: 0.60, 95% CI: 0.51–0.70	ROR: 0.79, 95% CI: 0.70–0.89
Social circumstances	360	NA	NA	NA	NA	NA	ROR: 2.94, 95% CI: 2.27–3.81
Surgical and medical procedures	333	ROR: 1.06, 95% CI: 0.76–1.48	NA	ROR: 1.41, 95% CI: 0.72–2.75	ROR: 2.12, 95% CI: 1.16–3.88	ROR: 1.28, 95% CI: 0.95–1.72	ROR: 3.07, 95% CI: 2.34–4.03
Vascular disorders	1127	ROR: 0.57, 95% CI: 0.49–0.66	ROR: 0.56, 95% CI: 0.46–0.69	ROR: 0.35, 95% CI: 0.27–0.43	ROR: 0.35, 95% CI: 0.29–0.41	ROR: 0.26, 95% CI: 0.23–0.29	ROR: 0.64, 95% CI: 0.58–0.70

Table S5. Quantitative disproportionality patterns by PT (Classification based on Reporting Odds Ratio and 95% confidence interval)

DDI cases	Drug interaction	Inhibitory drug interaction	Labelled drug-drug interaction medication error	Off label use	Product use in unapproved indication	Accidental overdose	Overdose	Intentional overdose	Product dose omission issue	Underdose	Incorrect dosage administered	Incorrect dose administered	Wrong dose
Digoxin	833	5	14	113	48	166	573	94	9	3	11	39	12
Dobutamine	17	0	0	75	30	0	6	4	12	1	0	3	0
Milrinone	5	0	0	26	12	6	4	0	1	0	1	3	0
Levosimendan	1	0	0	12	9	3	6	0	0	1	1	4	0
Dopamine	24	0	0	28	7	3	16	10	1	0	0	5	1
Epinephrine	88	5	7	190	74	104	85	50	159	15	3	65	4
Norepinephrine	24	0	2	77	64	21	48	39	11	3	6	18	6
